# Dissociated brain functional connectivity of fast versus slow frequencies underlying individual differences in fluid intelligence: a DTI and MEG study

**DOI:** 10.1038/s41598-022-08521-5

**Published:** 2022-03-18

**Authors:** S. E. P. Bruzzone, M. Lumaca, E. Brattico, P. Vuust, M. L. Kringelbach, L. Bonetti

**Affiliations:** 1 Center for Music in the Brain, Department of Clinical Medicine, Aarhus University & The Royal Academy of Music Aarhus, Aalborg, Denmark; 2 Centre for Eudaimonia and Human Flourishing, Linacre College, University of Oxford, Oxford, UK; 3 Department of Psychiatry, University of Oxford, Oxford, UK; 4 Department of Education, Psychology, Communication, University of Bari Aldo Moro, Bari, Italy; 5 Neurobiology Research Unit (NRU), Rigshospitalet, Copenhagen, Denmark

**Keywords:** Neuroscience, Cognitive neuroscience, Intelligence

## Abstract

Brain network analysis represents a powerful technique to gain insights into the connectivity profile characterizing individuals with different levels of fluid intelligence (G*f*). Several studies have used diffusion tensor imaging (DTI) and slow-oscillatory resting-state fMRI (rs-fMRI) to examine the anatomical and functional aspects of human brain networks that support intelligence. In this study, we expand this line of research by investigating fast-oscillatory functional networks. We performed graph theory analyses on resting-state magnetoencephalographic (MEG) signal, in addition to structural brain networks from DTI data, comparing degree, modularity and segregation coefficient across the brain of individuals with high versus average G*f* scores. Our results show that high G*f* individuals have stronger degree and lower segregation coefficient than average *Gf* participants in a significantly higher number of brain areas with regards to structural connectivity and to the slower frequency bands of functional connectivity. The opposite result was observed for higher-frequency (gamma) functional networks, with higher G*f* individuals showing lower degree and higher segregation across the brain. We suggest that gamma oscillations in more intelligent individuals might support higher local processing in segregated subnetworks, while slower frequency bands would allow a more effective information transfer between brain subnetworks, and stronger information integration.

## Introduction

A fundamental characteristic of the human brain is the plethora of different cognitive abilities that allow us to flexibly adapt to the environment^1–3^. Among these, intelligence has captured the attention of multiple research domains^4–8^. According to the classification by Cattell^4^, general intelligence (G) can be divided into fluid (G*f*) and crystallized (G*c*) intelligence, which are present across the population with measurable inter-individual differences^1,5^. While G*c* reflects the previously learned procedures and acquired knowledge, G*f* relates to processes such as abstract and logical reasoning and visuo-spatial problem-solving^1,2,5^, only minimally depending on prior learning and acculturation^4^, and is relatively stable across the lifespan^8^. Typical tasks measuring G*f* correspond to figure analyses and classifications, mental manipulation of series of numbers and letters, and visuo-spatial matrices^4^.

The neural underpinnings of G*f* have been extensively studied by means of different techniques for data acquisition and analysis and various psychometric tests and tasks^5,9–16^. In this respect, one of the most accredited theories on the neural basis of G is the Parieto-Frontal Integration Theory of intelligence (P-FIT)^17,18^. According to this theory, cognitive performance arises from a hierarchical chain of subsequent brain processes. Here, incoming sensory information from temporal and occipital areas is first elaborated in parietal regions and subsequently integrated and abstracted in the frontal areas of the brain.

Although the P-FIT theory is intriguing and well-posed, its approach tends to localise the main brain areas involved in cognitive processes instead of directly considering the brain as a holistic dynamic network where the resolution of complex cognitive tasks relies on constant communication across the wholebrain. Indeed, recent research supports the fact that the brain should be considered as a dynamic network and its properties studied as such^19,20^. In this framework, an optimal efficiency of information flow would be favoured by a balanced ratio between segregation (i.e., processing of information in local subnetworks) and integration (i.e., processing of information linking different subnetworks through long-range connections)^19,20^.

Coherently with this perspective and with the P-FIT theory, previous studies showed specific differences of white matter structure and connectivity patterns in the brain of participants scoring higher in G*f* tasks. In particular, studies using diffusion tensor imaging (DTI) reported an association between greater white matter integrity in the superior longitudinal fasciculus, an association tract connecting frontal, parietal, temporal and occipital lobes, and greater G*f* scores as measured with the Weschler Adult Scale of Intelligence (WAIS)^21–24^. In addition, network analysis of DTI-derived structural connectivity using graph theory measures showed higher global efficiency and shorter characteristic path length in participants with high versus average G*f* scores^24,25^, supporting the contribution of correctly balanced integration and segregation processing to G*f* abilities.

In accordance to the anatomical findings, a growing body of evidence based on lesion^26–28^ and functional magnetic resonance imaging (fMRI) studies pointed at a close link between G*f* and a specific subset of brain regions behaving as hubs within the whole-brain network^12,29,30^. This set of dynamically interacting areas, involving bilateral temporal, parietal and frontal regions, forms what is also referred to as “multiple demand” (MD) network^12,29,30^ and provides an example of the need for signal integration across spatially segregated brain areas in G*f*.

Along this line, former findings from electroencephalography (EEG) studies pointed toward an optimized brain network configuration in individuals with greater G*f* scores and a key role of the parietal and frontal  cortices within such network^31,32^, coherently with both the P-FIT and the MD network theories. However, only a limited number of studies explored the functional brain networks of G*f* using graph theory and real-time neurophysiological measurements such as EEG, while no study to date has used magnetoencephalography (MEG). Given the fast-scale dynamics of brain activity, investigating the brain networks underlying G*f* with such methods would provide more accurate insights about the neurophysiological underpinnings of G*f*. Moreover, little is known about the relationship between functional connectivity in different frequency bands and individual variation of G*f*.

Thus, in this study, we used for the first time MEG to explore the differences in the brain networks of high versus average G*f* individuals as emerging from fast-scale whole-brain functional connectivity. Based on resting-state neural activity (rs-MEG), we computed functional connectivity within five main frequency bands (delta: 0.1–2 Hz, theta: 2–8 Hz alpha: 8–12 Hz, beta: 12–32 Hz, gamma: 32–75 Hz) and investigated the properties of the fast-scale networks with graph theory measures. In line with previous research, we also explored the organization of the anatomical networks based on DTI images. We hypothesized to observe a different network organization in participants' brains characterized by a high versus average G*f*. With regards to structural connectivity, according to previous literature^22,24^, we expected to detect a higher proportion of long-range connections as well as a higher inter-subnetwork connectivity for the high versus average G*f* group. Regarding the rs-MEG signal, we hypothesized to observe different results across the five frequency bands. Specifically, since previous studies showed the importance of slow brain rhythms for long-range communications^33–35^, we expected to detect a higher proportion of long-range and inter-subnetwork functional connections among slow bands in high versus average G*f* participants. Conversely, based on the established role of fast frequencies for local connectivity^33,34^, we hypothesized to observe a higher level of intra-subnetworks communication among gamma band in high versus average G*f*s.

## Results

### Experimental design

In this study, we aimed to characterize the neural correlates of fluid intelligence by using graph theory measures on functional and structural connectivity. Specifically, we were interested in measures indexing connectivity of each brain ROIs with the rest of the brain and returning an estimation of the intra- and inter-subnetworks connectivity. Furthermore, we wished to investigate whether high versus average G*f* participants presented different community structures. For these reasons, we mainly focused on degree, modularity, and segregation coefficient.

We acquired structural DTI using MRI and measured brain activity with MEG during 10 min of resting state with eyes open. Next, we collected behavioural measures of intelligence using the Wechsler Adult Intelligence Scale IV (WAIS-IV). The experimental procedures involved a total of 71 participants who gave their informed consent, but two participants had to be excluded since they did not perform the WAIS-IV tests. Our 69 WAIS-IV participants were divided into two groups based on their mean G*f* and by considering at least one standard deviation (std; standardized WAIS-IV std = 15) apart, so that the distinction between the two groups was psychometrically meaningful, as suggested by previous literature on the topic^36–39^. The resulting groups were labelled as high G*f* (N = 38; mean G*f* = 117.72 ± 4.66) and average G*f* (N = 31; mean G*f* = 102.98 ± 6.09). As expected, the difference between the two groups was significant on a statistical level (t-test: *p* < 1.0e−07, *t*(55) = 11.08) (See “Methods” for further background and statistical information on the two groups). Finally, since we had to discard a few participants due to technical problems during the acquisition of DTI and MEG data, our final sample for WAIS-IV and DTI analysis consisted of 67 participants, while the one for the WAIS-IV and MEG analysis of 66 participants.

### Data analysis overview

Based on the non-cerebellar parcels of the automated anatomical labelling (AAL) brain parcellation, we constructed functional and structural connectivity matrices for each participant. The structural connectivity matrix was created based on the probabilistic tractography computed across all the 90 AAL regions of interest (ROIs) of the DTI images. The functional connectivity matrix was realized after reconstructing the sources of the MEG brain signal,  by using the widely adopted solution named beamforming^40^ (see “Methods” for details) in AAL space. Then, we estimated functional connectivity by computing Pearson’s correlations between the envelope of the timeseries of each pair of the 90 AAL brain areas. These correlations have been computed across the whole duration of the MEG recording (approximately 10 min). Importantly, the functional brain data was reconstructed in five different frequency bands (delta: 0.1–2 Hz, theta: 2–8 Hz alpha: 8–12 Hz, beta: 12–32 Hz, gamma: 32–75 Hz), returning a rather complete picture of the fast-scale information flow in the brain during resting state. Next, we computed graph theoretical measures of the individual brain structural and functional networks and compared them between the two groups of participants (high versus average G*f*).

**Figure 1:**
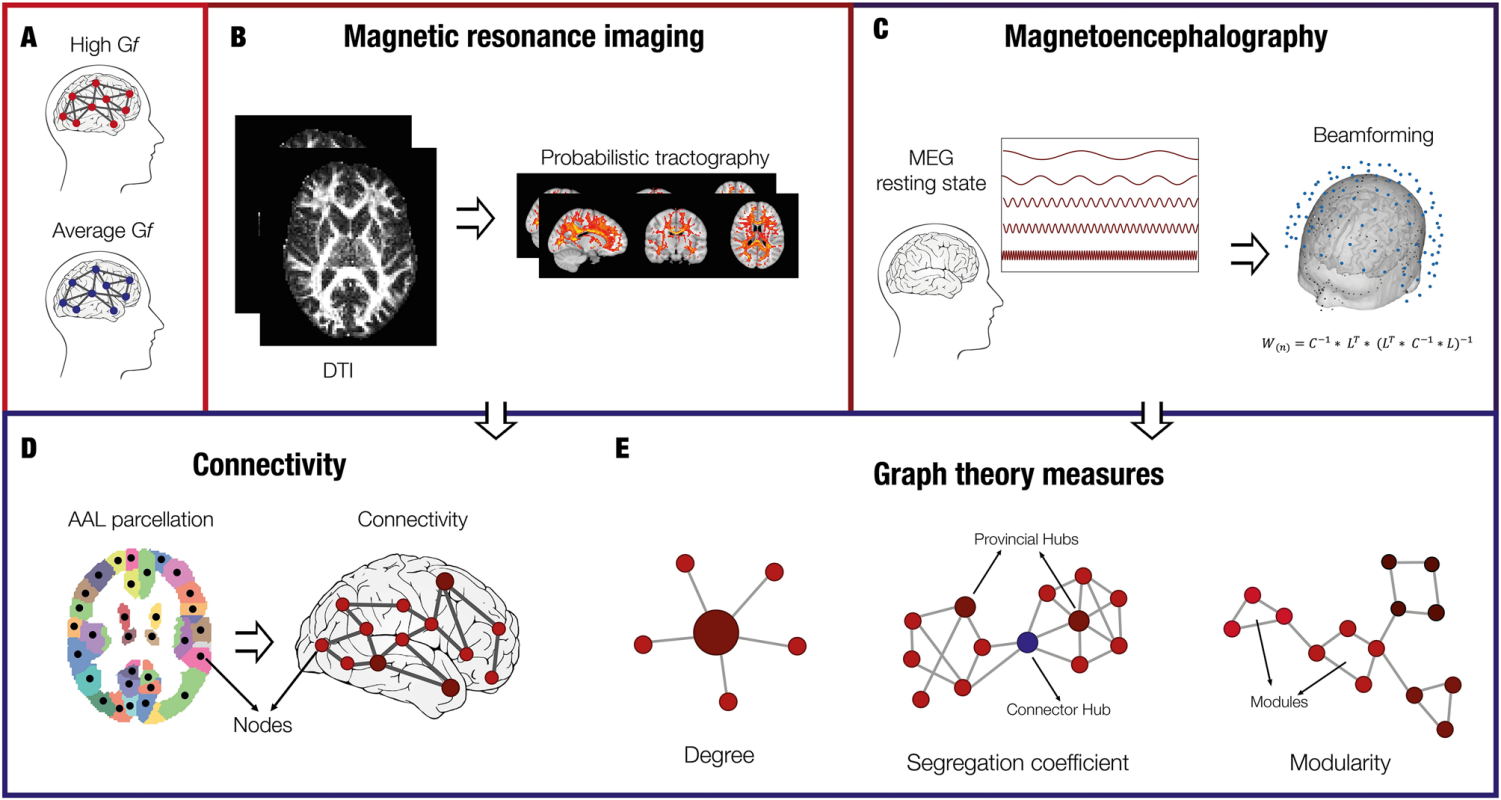
Experimental design and analysis pipeline. (**A**) Participants were divided into two experimental groups, namely average Gf and high Gf, based on their scoring to perceptual reasoning, working memory, and speed processing indexed by WAIS-IV. (**B**) Diffusion-tensor imaging (DTI) data were collected and pre-processed for both groups. Then, the white matter bundles were modelled using probabilistic tractography. (**C**) For both groups, magnetoencephalographic (MEG) data were collected during a 10-min session of resting state. The data were filtered to analyse five different frequency bands: 0.1–2 Hz (delta), 2–8 Hz (theta), 8–12 Hz (alpha), 12–32 Hz (beta), 32–74 Hz (gamma). Next, they were source-reconstructed with the beamforming algorithm. (**D**) Connectivity was computed for both DTI and MEG data for each subject. The connectivity matrix for the DTI data was created by computing the probabilistic tractography based on AAL parcellation. The connectivity matrix for MEG data was estimated by computing the Pearson’s correlations between the envelope of each pair of brain areas timeseries. (**E**) Graph measures were used to investigate the structural and functional brain networks of each group. Degree, modularity, and segregation coefficient provided the most insightful results.

Specifically, we were interested in the brain organization in terms of ROIs degree, segregation in different subnetworks (communities) and intra- and inter-subnetworks connectivity. Moreover, we aimed to detect how the brains of high versus average G*f* participants were organized in terms of structural connections and fast-scale information flow during resting state. The overview of the analysis pipeline is illustrated in Fig. 1.

### Structural connectivity

**Figure 2:**
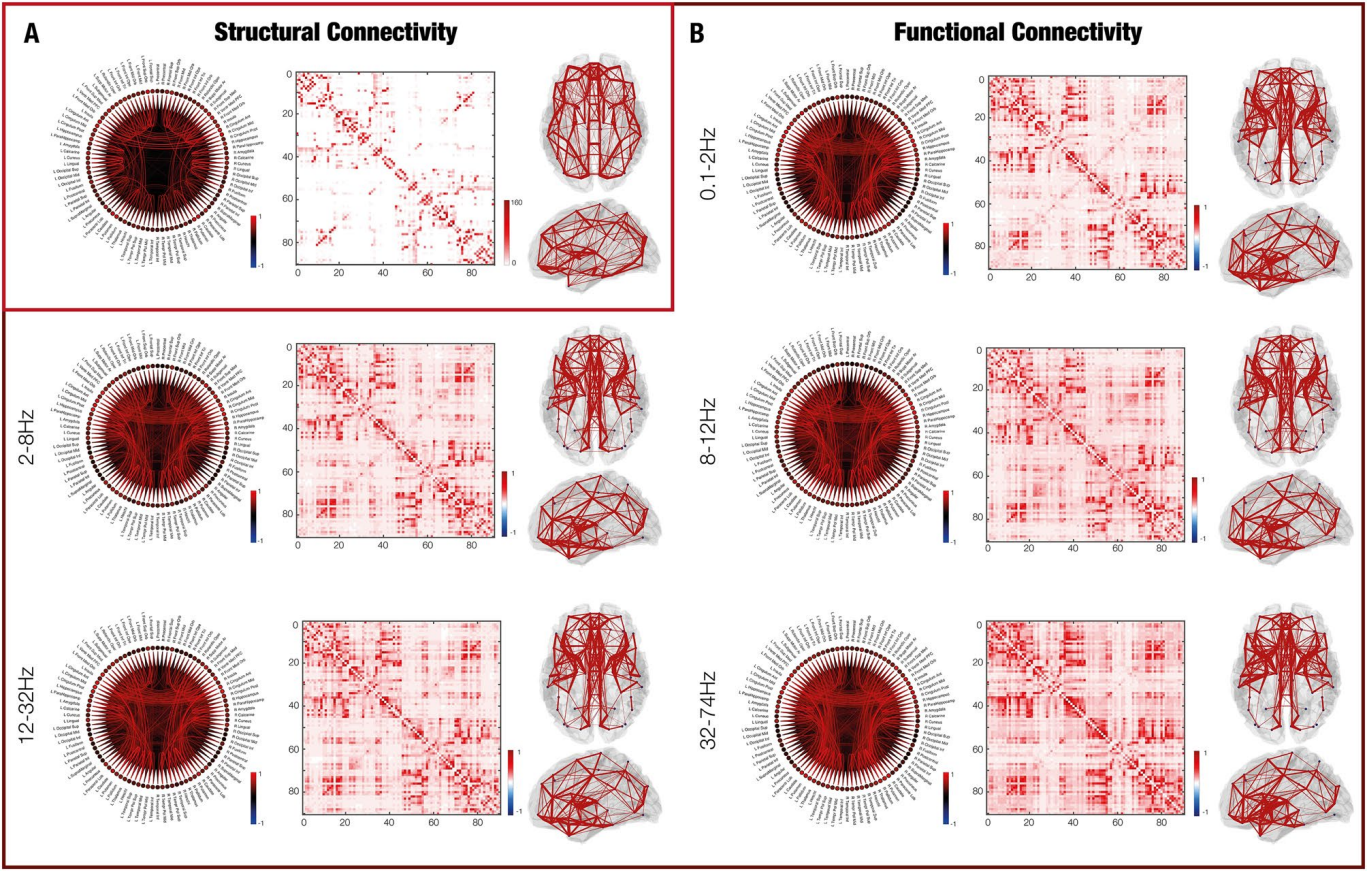
Structural and functional whole-brain connectivity. (**A**) Structural connectivity computed from DTI data. The circular connectogram and the connectivity matrix represent the connections between the 90 AAL nodes. The different connection strengths are represented by different colour shades. The whole-brain figures depict the whole-brain connections, with stronger connections being thicker. Colourbars indicate the normalized average number of streamlines connecting the brain areas. (**B**) Similarly, functional connectivity computed from MEG data, for each of the five frequency bands analysed. Colourbars indicate the Pearson’s correlations, showing the functional connectivity between brain areas.

After pre-processing the DTI data, matrices of structural connectivity were constructed for every participant using the output of the probabilistic tractography, which was normalized for the size of the brain ROIs (see “Methods” for details). We constrained the structural matrices to the non-cerebellar parcels of AAL parcellation (where each of the 90 regions represented a node of the brain network), resulting in a 90 × 90 matrix. The structural connectivity averaged across participants is shown in Fig. 2A.

### Functional connectivity

Individual functional connectivity matrices were constructed based on the pre-processed and source reconstructed MEG data, for each of the five frequency bands considered in the study: delta, theta, alpha, beta and gamma. As done for the DTI data, the reconstructed neural signal was constrained to the 90 non-cerebellar AAL parcellation. The resulting 90 × 90 matrix contained the information regarding the correlations between the 90 AAL brain regions, where each region represented a node of the brain network. The average functional connectivity across participants is shown in Fig. 2B, independently for each frequency band.

### ROIs degree

We analysed the two types of connectivity using graph theory measures between participants who scored high versus average in the WAIS-IV.

**Figure 3:**
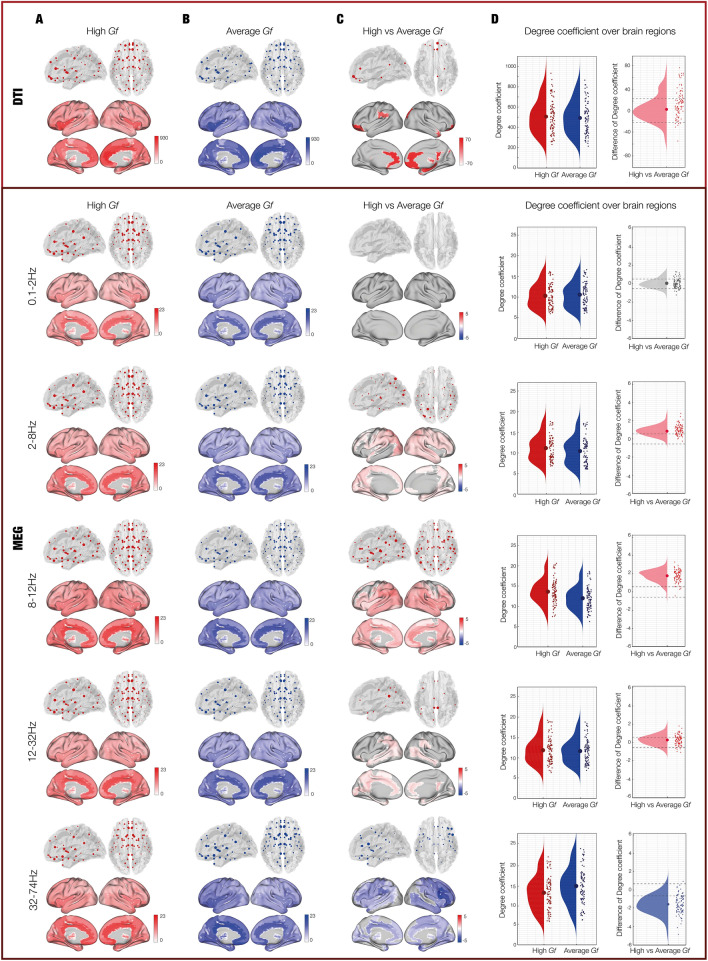
Degree of connectivity. (**A**) Degree coefficients of structural and functional connectivity in participants with high Gf. (**B**) Degree coefficients of structural and functional connectivity in participants with average Gf. (**C**) Contrasts of the degree coefficients between the two groups. In the contrast, the red colour indicates that high Gf individuals had stronger degree among a significantly higher number of ROIs, while blue showed a stronger degree among a significantly higher number of ROIs for average Gf participants. This column illustrates the ROIs whose degree coefficients were stronger than at least one standard deviation above the mean across all ROIs. (**D**) Degree depicted for every brain ROI of high, average and high versus average Gf. Each dot shows the degree of each of the 90 ROIs, independently for high and average Gf participants. Dashed lines indicate the standard deviation with reference to zero, helping to identify whether the ROIs had a stronger or weaker degree for high versus average Gf participants.

First, we investigated whether the degree of the ROIs across the whole-brain differed among the two G*f* groups, after having verified that the variances of the two groups were not significantly different (*p* > 0.05). Participants belonging to the high versus average G*f* group showed significantly higher ROIs degree in both structural (*p* = 0.007) and functional networks for theta (*p* < 0.001), alpha (*p* < 0.001) and beta (*p* = 0.004) frequencies, indicating an overall stronger level of connectivity between ROIs for the high G*f* participants (Fig. 3). Remarkably, the ROIs where this difference was mainly marked for structural connectivity and theta, alpha and beta frequency bands were provided bilaterally by a widespread network involving frontal (postcentral gyrus, superior frontal gyrus, postcentral gyrus, supplementary motor area), parietal (inferior and superior parietal lobule), occipital regions (inferior, middle and superior occipital gyrus) and temporal (middle and superior temporal gyrus) regions, as well as multiple subcortical areas (parahippocampal gyrus in the structural and in the functional, hippocampus, cingulum, thalamus in the functional). Conversely, individuals with average G*f* scores showed greater ROIs degree across the whole-brain than the high G*f* participants for the gamma frequency (*p* < 0.001). In this case, stronger degree centrality was observed in frontal, medio-temporal and subcortical areas, regions that greatly overlap to those that were more central for high versus average G*f* scores. A detailed list of the most central regions and the correspondent degree coefficients in structural and functional brain networks in the two experimental groups can be found in Table ST1. No significant difference was found for delta frequency band.

### Community structure and modularity

First, we estimated the community structure and modularity (depicted in Fig. 5 and reported in Table ST2) using the modularity algorithm introduced by Newman^41^. This procedure assumes that modularity can be expressed in terms of the eigenvectors of the characteristic matrix for the network, which Newman named the modularity matrix. Such procedure allows to detect a community structure of the brain network consisting of a subdivision of non-overlapping subnetworks of nodes (brain ROIs) that maximizes the number of within-group connections and minimizes the number of between-group connections (Fig. 5B–F). Modularity refers to a statistic able to quantify the degree to which the network can be divided into clearly delineated subnetworks. Newman’s algorithm is widely adopted in network analysis of the brain and returns results of demonstrably higher quality than competing methods. Also, it is very fast to compute^41^.

Here, we computed the modularity of the brain networks at the group-level, independently for the two experimental groups (high and average G*f*). Then, using MCS we tested the modularity values of structural and functional connectivity matrices (for the five frequency bands independently) against chance, to detect whether the brain networks were more inclined to be divided into subnetworks (more divisible into subgroups) than random configurations of the same original brain networks. The test was significant for both structural and functional connectivity matrices (*p* < 0.001).

### Segregation coefficient

**Figure 4:**
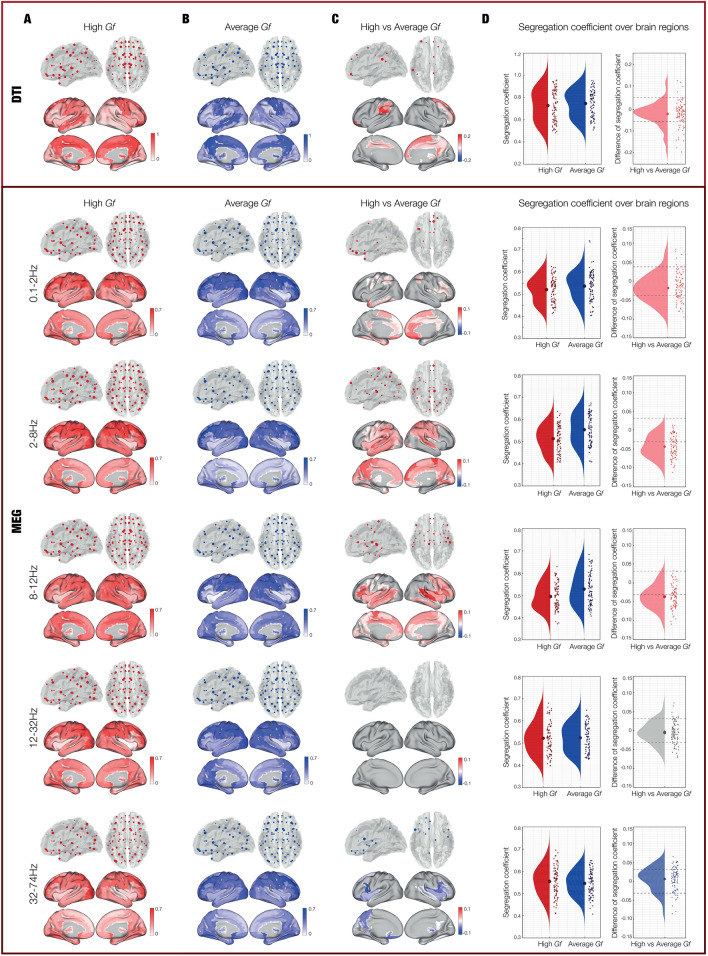
Segregation coefficient. (**A**) Segregation coefficient computed from structural and functional connectivity in participants with high Gf. (**B**) Segregation coefficient computed from structural and functional connectivity in participants with average Gf. (**C**) Contrasts related to the segregation coefficient between the two groups. In the contrast, the red colour indicates that high versus average Gf individuals had a weaker segregation coefficient among a significantly higher number of ROIs, meaning that they presented more inter-subnetwork connections. Conversely, the blue colour showed that average versus high Gf individuals had a weaker segregation coefficient among a significantly higher number of ROIs, meaning that they presented more inter-subnetwork connections. (**D**) Segregation coefficient of high, average, and high versus average Gf participants. Here, each dot shows the segregation coefficient of each of the 90 ROIs, independently for high and average Gf participants. Dashed lines indicate the standard deviation with reference to zero, helping to identify whether the ROIs had a stronger or weaker segregation coefficient for high versus average Gf participants.

Then, we computed the segregation coefficient and compared it between the G*f* groups. This coefficient, ranging from zero to one, shows the level of connectivity of a ROI with the ROIs belonging to the same community when tending to one, or to ROIs of other communities when tending to zero. Here, we studied the ROIs segregation coefficient over the whole-brain in the high versus average G*f* participants, after having verified that the variances of the two groups were not significantly different (*p* > 0.05). The results showed that high versus average G*f*s presented higher ROIs segregation coefficient for both structural connectivity (*p* < 0.001) and delta (*p* < 0.001), theta (*p* < 0.001) and alpha (*p* < 0.001) bands of the functional networks (Fig. 4). The ROIs with the strongest segregation coefficient in these frequencies were found bilaterally in parietal, temporal, cingulate and subcortical areas (see Table ST3). Conversely, ROIs with the lowest segregation coefficient were found in participants with average versus high G*f* for the gamma frequency band (*p* = 0.003), mainly in frontal, temporal and subcortical regions (Table ST3. No differences were found between the two groups for the functional connectivity in beta frequency band.

### Modularity, density, characteristic path length, global and local efficiency

Modularity, density, characteristic path length, global and local efficiency were not significantly different between the two groups, neither in the structural nor in the functional networks (*p* > 0.002).

### Integration between structural and functional connectivity and G*f*

We have carried on an analysis to assess whether we could combine our two modalities (SC and FC) and study such combination in light of the G*f* differences.

We computed structural and functional connectivity matrices and correlated them independently for each participant and frequency band. Afterwards, we grouped the participants into our two experimental groups (high and average Gf) and tested with an ANCOVA (considering age, sex, and education as covariates) whether the two groups differed in terms of similarity between structural and functional connectivity. The ANCOVA was not significant (*p* > 0.05).

## Discussion

In this study, we investigated the structural (DTI) and functional connectivity (rs-MEG) differences in individuals with high versus average G*f* scores. We found  a stronger degree for both structural connectivity and the slower frequency bands of functional connectivity in high compared to average G*f* individuals. On the contrary, gamma band presented stronger degree of brain areas for average versus high G*f*. Then, based on the estimation of the community structure, we computed the segregation coefficient. Brain areas of high versus average G*f*s presented a different community structure and a lower segregation coefficient for structural connectivity and for the slower frequency bands of functional connectivity, and a higher segregation coefficient for gamma band.

### Structural connectivity and G*f*

After assessing that the computation of our structural and functional connectivity matrices (illustrated in Fig. 2) returned results coherent with previous literature^42–46^, we investigated them in relation to G*f.*

Our results for structural connectivity indicated that higher versus average G*f* participants presented stronger long-range and inter-subnetwork connectivity, as reflected by the smaller values of segregation coefficient. These findings supported previous studies which reported associations between G*f* and anatomical connectivity in the brain. For example, FA measured in the superior longitudinal fasciculus was linked to greater scores in the Weschler Adult Scale of Intelligence (WAIS) for the G*f* tasks^22^. Since the superior longitudinal fasciculus is an association tract that connects frontal, parietal, temporal and occipital lobes, our results support the perspective that higher G*f* individuals have stronger association and long-range connections. Moreover, Li and colleagues^24^ studied topological properties of the brain networks using graph theory in participants with high and general IQ scores derived from the Chinese version of the WAIS test. Brain networks of high versus general IQ participants had higher global efficiencies and shorter characteristic path length, which authors interpreted as a more efficient parallel transfer of the information in the brain. Although we did not find significant differences in characteristic path length or in global efficiency, our results about degree, modularity and segregation coefficient are overall coherent with those by Li and colleagues. Furthermore, the IQ measures used by Li and colleagues^24^ involved not only G*f*, but also G*c* tasks, suggesting that such topological measures may be more relevant when approaching intelligence from a broader perspective.

To summarize, on the one hand our work confirmed the main findings provided by previous studies, showing that high versus low/average intelligent individuals have a different network organization, especially whit regards to long-range connections. On the other hand, our study integrated previous research by highlighting the specific role of degree, modularity, and segregation coefficient in characterizing the difference between high versus average G*f* people.

### Functional connectivity

The main novelty of the current work consisted of investigating differences between high versus average G*f* in relation to five frequency bands emerging from the fast-scale connectivity computed from rs-MEG.

Indeed, differently from our work, previous studies on functional brain networks and intelligence focused on brain lesion^26–28^ and fMRI. Taken together, they highlighted a subset of brain regions that arguably represented key hubs for the functional substrate of human intelligence^12,29,30^, comprising bilateral temporal, parietal and frontal regions of the brain, sometimes described as the MD network^12,29,30^. Furthermore, van den Heuvel and colleagues^25^ computed topological graph theoretical measures on fMRI data, reporting positive correlations between intellectual performance and the global efficiency of functional brain networks. Finally, a few studies connected G*f* and functional brain networks using EEG^31,32^, suggesting that individuals with greater G*f* scores presented a more optimized brain network configuration.

Although this research advanced our knowledge on the functional organization of the brain of intelligent individuals, previous evidence on the fast-scale functional connectivity of the brain in relation to G*f* remained scarce. Furthermore, no studies used MEG nor showed differences among frequency bands when investigating the neural underpinning of G*f*. Conversely, our research presented a different relationship between G*f* and functional organization of the brain networks when investigating a very fast frequency like gamma or slower frequencies such as delta, theta, alpha, and beta. Indeed, our results suggested that the functional resting state network in gamma frequency presented more intra-subnetwork connectivity and thus arguably more segregation and less information flow across the whole-brain in high versus average G*f*. Conversely, higher G*f* individuals may present a stronger integration between brain subnetworks and thus more long-range integration of information among slower frequency bands.

Such findings are coherent with previous literature proposing gamma band for local communication of brain areas and short-distance information flow^33,34^ and slower rhythms such as alpha and theta for long-range functional connections and communications between brain areas far away from each other^33–35^. In this perspective, we argue that the investigation of the brain network configuration of fast and slow frequency bands is of great importance to properly characterize and understand the neural substrate of G*f* and integrate previous knowledge, mainly derived from DTI and fMRI studies.

Finally, our analyses returned a different organization of the brain subnetworks in high versus average G*f*. As expected, overall, these subnetworks grouped together brain regions within frontal and temporo-occipital lobes, independently for the two hemispheres and coherently with previous literature^42,43,46^. Notably, the assignment of the cingulate gyrus to a brain subnetwork differed between high and average G*f*, highlighting the structural and functional integration of such brain area within frontal subnetworks of the brain in high G*f*s. Conversely, in the average G*f* the cingulate was segregated in an independent module for the structural connectivity and less connected to the frontal subnetworks for the functional connectivity. Although these results do not represent the focus of our work, they provide further evidence of the difference between the brain network organization of high and average G*f* and may be further explored by future investigations.

## Conclusions

Altogether, our findings point to a different whole-brain configuration of connectivity between individuals with high versus average G*f*. While our DTI findings confirm and support previous literature about structural connectivity, the MEG results integrate previous knowledge on the brain network organization among slower and faster frequency bands. Future studies are called to further investigate such phenomenon and provide additional evidence about the brain mechanisms underlying integration and segregation of the information across brain subnetworks and their relationship with G*f*.

Moreover, in our study, we reported network metrics independently derived from both structural and functional connectivity. In addition, we carried out one correlational analysis which combined the two modalities and investigated them in relation to G*f*. Although this analysis did not return significant results, we believe that more elaborated approaches might. Thus, future research is called for to conduct deeper investigations on how the integration of structural and functional connectivity is reflected on high and average G*f* individuals. For instance, whole-brain computational modelling of functional connectivity might be performed based on the structural connectivity and then compared between high versus average G*f*s.

Finally, while in our study we used solid and well-established metrics for computing functional connectivity such as Pearson’s correlations of the envelope of the MEG signal, future research may use different measures of connectivity (e.g. instantaneous phase, moving windows) to investigate the relationship between G*f* and dynamic measures of functional connectivity brain networks.

## Methods

### Participants

We recruited a total of 71 healthy participants, 35 females and 36 males (aged 18–42, mean age: 25.06 ± 4.11 years) of different nationalities. Two participants had to be excluded since they did not perform the WAIS-IV tests. Further, for the DTI data, two participants were excluded from the sample due to the poor quality of the data, after the computation of the pre-processing pipeline. Thus, the final sample for DTI consisted of 67 healthy volunteers (34 females, 33 males, mean age: 24.94 ± 4.05 years). Regarding MEG, three participants were excluded because it was not possible to record their MEG resting state data. Thus, the final sample for the MEG functional connectivity analyses consisted of 66 healthy volunteers (34 females, 32 males, 24.95 ± 4.24 years). Participants were recruited on a voluntary basis and compensated with vouchers. They were healthy and not under any medication. Furthermore, they did not report any neurological or psychiatric problems occurred in their past.

All the experimental procedures were approved by the Ethics Committee of the Central Denmark Region (De Videnskabsetiske Komitéer for Region Midtjylland) (Ref 1-10-72-411-17), in compliance with the declaration of Helsinki—Ethical Principles for Medical Research. Moreover, informed consent has been obtained from all participants before starting with the experimental procedures.

### Experimental design and overview of the analysis pipeline

In this study, we aimed to investigate whether structural and fast-scale functional connectivity differed between participants characterized by high and average levels of fluid intelligence (G*f*).

Participants underwent the acquisition of functional (magnetoencephalography, MEG) and structural (magnetic resonance imaging, MRI) data. We recorded resting-state neurophysiological activity throughout 10 min of MEG recordings, during which participants were not engaged in any task and kept their eyes open. Regarding MRI, we acquired T1-anatomical and diffusion-weighted (DTI) brain images. Independently for each participant, we reconstructed the sources of the MEG signal by combining the MEG with the structural T1 MRI data in automated anatomical labelling (AAL)^43–45,47^ space and estimated the functional connectivity between each pair of non-cerebellar brain areas of AAL. Similarly, we computed individual structural connectivity matrices in AAL space^42,46^ based on the DTI images.

After acquiring the neuro-functional and -structural data, we collected behavioural measures to estimate the participants’ G*f* along the following main scales of the fourth edition of the Wechsler Adult Intelligence Scale (WAIS-IV)^48^: perceptual reasoning, working memory and speed processing. All the tests were carried out in English, which was spoken fluently as a second language by the participants.

Finally, as described in the following paragraphs, we used graph theory measures to analyse group differences between high versus average G*f* in both structural and functional brain networks.

### Participants’ *Gf* scores

The mean G*f* score across the 69 (WAIS-IV subsample), 67 (WAIS-IV and DTI subsample) or 66 (WAIS-IV and MEG subsample) participants was nearly identical (111.10 ± 9.09; 111.45 ± 9.13 and 110.76 ± 9.05, respectively). Thus, the following numerical information about the two G*f* groups (Table 1) that we have used in our experiment will be reported for the full sample of 69 participants who were administered the WAIS-IV. Indeed, our sample was divided in two groups based on their mean G*f* and by considering at least one standard deviation (standardized WAIS-IV std = 15) apart, so that the distinction between the two groups was psychometrically meaningful, as suggested by previous literature on the topic^36–39^. This procedure yielded two groups: the high G*f* group (N = 38; mean G*f* = 117.72 ± 4.66); the average G*f* group (N = 31; mean G*f* = 102.98 ± 6.09). As conceivable, the difference between the two groups was also statistically significant (*p* < 1.0e−07, *t*(55) = 11.08). Importantly, we controlled that the two groups were matched in terms of socio-economical, demographic, and educational status. In both groups, participants were mainly of Danish nationality and all of them came from a Western cultural country. The High G*f* group comprised 15 females and 23 males with an average age of 25.86 ± 4.89. The Average G*f* group comprised 18 females and 13 males with an average age of 24.00 ± 2.69. The age difference was not significant (*p* = 0.05). Furthermore, the mean of the education years was 14.73 ± 4.25 for the high G*f* and 14.56 ± 5.87 for the average G*f*. Neither this difference was significant (*p* = 0.37).

**Table 1:** Participants’ demographic data.

High *Gf* (n = 38)					Average *Gf* (n = 31)				
Age	*Gf*	Years of education	Handedness	Sex	Age	*Gf*	Years of education	Handedness	Sex
25.86 ± 4.89	117.72 ± 4.66	14.73 ± 4.25	3 left-handed	15F; 23 M	24.00 ± 2.69	102.98 ± 6.09	14.56 ± 5.87	1 left-handed	18F; 13 M

Demographic data of the participants divided into the two experimental groups. Age, Gf and years of education indicate means ± standard deviations.

### MEG data acquisition

We acquired both MRI and MEG data at the Aarhus University Hospital (Denmark) in two independent sessions. MEG data were acquired with a 306-channel (204 planar gradiometers and 102 magnetometers) Elekta Neuroimag TRIUX system (Elekta Neuromag, Finland), with a sampling rate of 1000 Hz and an analog filter of 0.1–330 Hz. Prior to the measurements, the head shape and spatial coordinates of each participant were digitalizaed with a 3D digitizer (Polhemus FastrakColchester, VT, USA). The head localization was determined using four Head Position Indicator coils (cHPI) that were registered with respect to three anatomical landmarks (fiducials), namely the nasion, left and right preauricular areas. The cHPI allowed to continuously track the head position in respect to the MEG sensors and to correct for head movements. Furthermore, the digitalization of the participants’ head provided the information for co-registering the functional data recorded by the MEG with the anatomical data acquired with the MRI.

### MRI data acquisition

Whole-brain T1-weighted and diffusion-weighted images were acquired with a Siemens Magnetom Skyra 3 T MRI scanner (20-channel head coils) located at Aarhus University Hospital, Denmark. T1 images were acquired with the following parameters: 1.0 × 1.0 × 1.0 mm voxel size (1.0 mm^3^); 256 × 256 reconstructed matrix size; 2.96 ms echo time (TE); 5000 ms repetition time (TR); 240 Hz/Px bandwidth. For the reconstruction of the MEG functional data, each T1-weighted scan was co-registered to the standard brain template from the Montreal Neurological Institute (MNI) using an affine transformation. Next, it was referenced to the MEG sensors space with the data about the head shape that was previously digitalized.

Diffusion-weighted images were acquired using echo-planar imaging (EPI), with the following parameters: 2.0 × 2.0 × 2.0 mm voxel size (2.0mm^3^); 104 ms TE; 3300 ms TR; 100 × 100 × 72 matrix size; 221 volumes in anterior–posterior (AP) direction; 1 volume in posterior-anterior (PA) direction; 2500 s/mm^2^ b-value; 29.41 Hz/Px bandwidth.

### DTI data pre-processing

We pre-processed the MRI diffusion data with the FMRIB’s Diffusion Toolbox (FDT) toolbox in the FMRIB Software Library (FSL)^49,50^. First, we visually checked the data to assess the good quality of the scans. After converting the files into *nifti* format, we created a reference volume (b0) based on the first image of both the AP and PA files, which we used to correct for susceptibility-induced distortions resulting in artefacts at the edge of the brain. Next, based on the corrected b0, we generated a brain mask that we applied to correct for head motion and eddy currents. In particular, eddy currents refer currents generated in the MRI machine because of the rapid change of the magnetic field direction during the acquisition (echo planar images are acquired rapidly in different orientations).

The pre-processed and corrected data were subsequently used for the estimation of the main white matter tracts with probabilistic tractography.

### Tractography in AAL

We modelled the whole-brain structural connectivity with the FSL probabilistic tractography for crossing fibres^51,52^, using the AAL parcellation in the MNI152 standard-space T1 weighted average image. First, based on the pre-processed data and the corrected reference volume b0, we estimated the fiber orientations of every voxel for each participant. Second, we created 90 seed masks—one for each AAL region—with voxels sized 2 × 2 × 2mm. Using a Markov Chain Monte Carlo algorithm, we estimated the probability distribution of fibre direction at each brain voxel, with 1000 fibres (streamlines) per voxel. Whole-brain tracts (structural connectivity between each pair of AAL brain regions) were estimated by considering the continuity between fibres of all the voxels contained in each AAL region and all the other AAL regions.

### Structural connectivity network

After the estimation of the probabilistic tractography, we have computed a few normalization steps to obtain a final structural connectivity matrix, one for each participant.

In our brain networks, the nodes were defined according to the AAL parcellation, with each non-cerebellar AAL parcel representing a node of the network. The networks that we computed were undirected (i.e. a → b = b → a). However, the FSL probabilistic tractography estimates independently the two directions of the connectivity between two nodes (i.e. a → b = b → a means the same, but are estimated with slightly different values). Thus, as previously done^42^, we averaged the two directions to obtain only one value of connectivity between any pair of brain areas and thus a truly symmetric undirected connectivity matrix. Finally, we have normalized each connection between AAL brain areas for the sizes of the same brain areas. This was done since larger AAL parcels may present more connections simply because they are larger and not because they are actually more densely connected. Thus, we have divided each connection between pairs of brain areas by the averaged size of those brain areas (e.g. a ↔ b/((size of a + size of b)/2)). The resulting 90 × 90 matrix represented an undirected, weighted brain structural network.

### MEG data pre-processing

For the first pre-processing steps of the raw MEG data, we used MaxFilter^53^. These steps consisted in applying signal space separation (SSS) to attenuate interferences originated outside the scalp, adjusting for head motion and down sampling the signal from 1000 to 250 Hz. Next, we converted the data into the Statistical Parametric Mapping (SPM) format and further proceeded with the analyses using the Oxford Centre for Human Brain Activity Software Library (OSL), a freely available toolbox that combines in-house-built functions with existing tools from FSL^49^, SPM^54^ and Fieldtrip^55^ working in the Matlab environment (MathWorks, Natick, Massachusetts, United States of America). The frequencies below 0.1 Hz, too low for being originated by brain activity, were removed with a high-pass filter. In addition, we applied a notch filter to correct for possible electric current-induced interferences and further down-sampled to 150 Hz. After visually inspecting the data, we removed the parts of the signal that were altered by large artefacts. Then, we performed independent-component analysis (ICA)^56^ to isolate and discard the artefacts generated by eyeblinks and heartbeat.

### Source reconstruction

The brain sources of the neural activity registered on the scalp by the MEG sensors were estimated by using the OSL implementation of the beamforming algorithm. Specifically, the forward solution was computed using an overlapping-spheres model in an 8-mm grid (comprising 3559 brain voxels). This solution represented a simplified geometric model of the MNI-co-registered anatomy of each participant, fitting a sphere separately for each MEG sensor^40^. Then, we performed the inverse solution by using a beamforming algorithm. Such procedure utilized a different set of weights sequentially applied to the source locations for isolating the contribution of each source to the activity recorded by the MEG sensors at each time-point^45,47^. Our beamforming computation was performed using both magnetometers and planar gradiometers.

Importantly, the source reconstruction was computed for five different frequency bands that were estimated after the ICA computation and subsequently reconstructed: delta: 0.1–2 Hz, theta: 2–8 Hz alpha: 8–12 Hz, beta: 12–32 Hz, gamma: 32–75 Hz.

### Functional connectivity network

After estimating the brain sources of the recorded MEG signal, we have computed one functional connectivity matrix for each participant, similarly to what we did for the structural connectivity based on the DTI data. First, the reconstructed functional data (3559 brain voxels) were constrained to the 90 non-cerebellar parcels defined by AAL. Next, we computed the envelope of the time-series from each brain region using the Hilbert transform. Finally, we estimated the functional connections between each pair of brain areas by computing Pearson’s correlations between the envelopes of the time-series of each pair of AAL brain regions^57^. The correlations have been computed across the whole recording of MEG resting state (approximately 10 min).

### ROIs degree of connectivity across the whole-brain

The degree of connectivity describes how connected a node is to the other nodes of the network and can provide information about the functional integration properties of the network. We computed the degree (*d*_*(n)*_) of node *n* (here, an AAL ROI) as the sum of the weighted connections of that node to all other nodes^20^. This provided us with a value for each ROI indicating its degree of connectivity, and thus its centrality within the whole-brain network. Note that we have used a weighted measure of degree (i.e. we computed the strength of the connections and not only a binary measure indicating whether the connections existed or not) to analyse connectivity without losing relevant information. Indeed, both structural and functional connectivity between two ROIs can be reliably described by a weighted value which provides more information that the binary information telling whether they are connected or not.

Since we were interested in the difference of ROIs degree among the whole-brain and not only considering a few specific ROIs, we did not test the ROIs independently, but we compared the overall difference of ROIs degree between high versus average G*f* participants. Specifically, first we used the Bartlett test to assess whether the variance within the two groups (high and average G*f*) was not significantly different. Second, we computed the difference between the median of the degree of each ROI for high versus average G*f* and tested whether such differences of medians were different from zero using MCS. If the ROIs degree among the whole-brain is similar/equal between the two groups, its difference will be approximately zero, with some ROIs slightly above zero and some others slightly below, by random chance. Conversely, if the degree is different between the two groups in most of the ROIs, at the higher rate than chance level, then such result indicates a relevant difference in terms of ROIs degree between the two groups. Thus, in our MCS, we tested whether the distribution of differences between high versus average G*f* ROIs degree was significantly different from zero. First, we computed the number of ROIs whose difference degree was higher and lower than zero. Then we permuted the original data across experimental groups and computed the difference between the median of ROIs degree for the two permuted G*f* groups and observed the distribution of the difference between the degrees with respect to zeros. We re-iterated this operation for 10,000 times, building a reference distribution of the difference between the ROIs degree in the permuted scenarios. Finally, we compared the original distribution of differences between high versus average G*f* ROIs degree with the permuted distribution. Since we tested the original distribution considering both tales of the permuted distribution (higher and lower than zero), the final MCS *p-value* was obtained by dividing the MCS α level by two (0.05/2 = 0.025). Similarly, for the degree of functional connectivity, we performed 10 statistical tests: one for each of the two tales of the reference distributions and for each of the five frequency bands considered in the study. Thus, we corrected for multiple comparisons using the Bonferroni correction, by dividing the MCS α level (0.05) by 10 (MCS *p-*value = 0.05/10 = 0.005).

### Modularity and community structure

**Figure 5:**
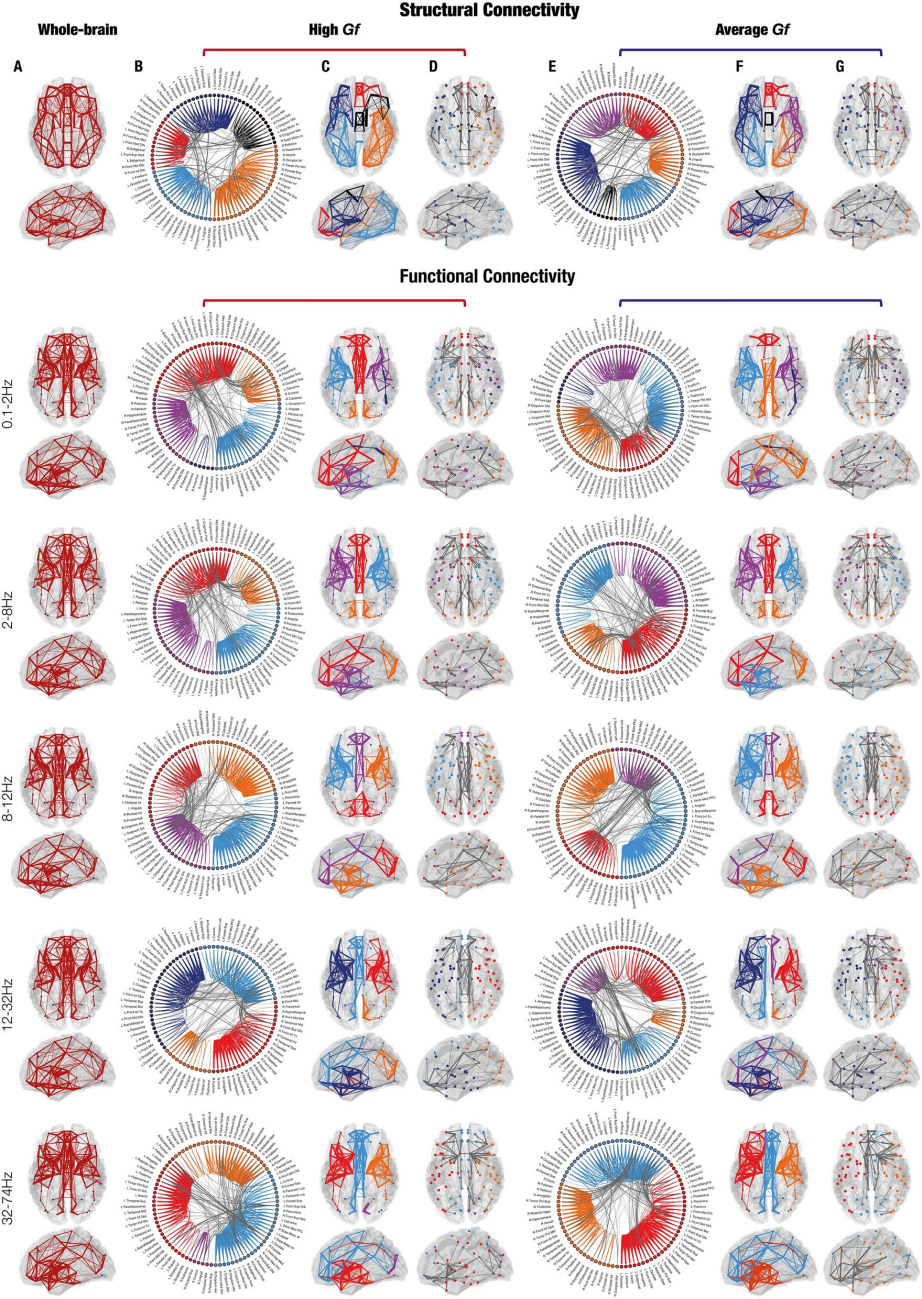
Inter and Intra-module connectivity in high versus average Gf. (**A**) Whole-brain structural and functional connectivity in all participants. (**B**) Circular connectogram representing inter- (in gray) and intra-module (different colors) connections in high Gf participants. (**C**) Brain modules and intra-module connections overlaid on a standard brain template, in individuals with high Gf. Different modules are represented by edges with different colors. (**D**) Inter-module connections in individuals with high Gf. Different modules are represented by dots in different colors, while inter-module connections are represented by grey edges. (**E**) Circular connectogram representing inter- (in gray) and intra-module (different colors) connections in average Gf participants. (**F**) Brain modules and intra-module connections in individuals with average Gf. Different modules are represented by edges with different colors. (**G**) Inter-module connections in individuals with average Gf. Different modules are represented by dots in different colors, while inter-module connections are represented by grey edges.

Modularity is a value describing the segregation of a network into discrete, non-overlapping clusters (modules) which optimize the network efficiency for specialized processing. In other words, it quantifies the degree to which a network can be subdivided into clearly defined, non-overlapping subnetworks. According to this definition, we computed the community structure by maximizing the intra-module connections within non-overlapping sub-modules of the network and minimizing the inter-module connections. To calculate this measure, we used the undirected measure of modularity developed by Newman implemented in the Brain Connectivity Toolbox (BCT)^20^, relying on the eigenvector solution^41^ and returning a discrete value of modularity and the corresponding community structure, representing the division of the AAL ROIs into distinct, non-overlapping subnetworks of the brain. While the community structure refers to a subdivision of the brain networks into non-overlapping subnetworks (Fig. 5), the modularity it a statistic able to quantify the degree to which the network can be divided into clearly delineated subnetworks. Newman’s algorithm is widely adopted in network analysis of the brain and returned results of demonstrably higher quality than competing methods and it is very fast to compute^41^.

Here, we computed the modularity of the brain networks at the group-level, independently for the two experimental groups (high and average G*f*). Then, we tested whether the modularity of the structural and functional brain data was significantly different by an equivalent network with connections placed randomly. To do so, we performed an MCS. First, we computed the modularity of the original data, corresponding to the averaged connectivity matrix (*M*) across participants. Second, we performed 1000 permutations of matrix *M* and extracted the modularity for the permuted data. This procedure yielded a reference distribution of permuted modularity values. Finally, we considered significant the original modularity value only if it was higher than the 99.9% of the permuted modularity values. This procedure was computed independently for the structural and functional data. A graphical depiction of the community structure for structural and functional brain networks is provided in Fig. 5 and reported in detail in Table ST2.

### Segregation coefficient

Based on the previously computed community structure, we were interested to observe whether the ROIs of high and average G*f* participants differed in terms of connectivity within and between the brain subnetworks. Specifically, we expected to find a tendency of high versus average G*f* individuals to have more pronounced connectivity between brain subnetworks. Thus, we computed a ratio that we referred to as “segregation coefficient”, which indicates whether an ROI is mainly connected to the other ROIs of the same subnetwork or is more connected to ROIs in other subnetworks. The coefficient is computed by dividing the degree of the *ROI a* with regards to the ROIs of the same subnetwork by the degree computed for *ROI a* with regards to all other ROIs (so also the ones of other subnetworks of the brain). Therefore, the coefficient values range between one and zero: the closer the coefficient to zero, the more the ROI has connections outside the community, highlighting its relevance as connector hub. Conversely, the closer the value to one, the greater the within-community degree, indicating that the ROI is mainly central within its own subnetwork. Note that this coefficient is very similar to the participation coefficient which captures the distribution of a node’s connections^58^. Indeed, the participation coefficient approaches one when a node has equal connections to all the subnetwork of a network. In our case, we used a slightly different measure (the segregation coefficient) since we were simply interested in evaluating the ratio of the connections between the node and its subnetwork and between the same node and every node of the brain network.

To test the difference of the whole-brain distribution of the segregation coefficient between high versus average G*f* individuals, we have performed an MCS analogous to the one described for the paragraph on the *Degree of connectivity*.

### Global measures of the brain graph

Although our focus was on degree, modularity, and segregation coefficient, we reported global measures of the brain graphs (structural and functional) to provide the readers with complete information.

#### Characteristic path length

The *Characteristic path length* represents the average shortest path length between all pairs of nodes composing the network (e.g. the minimum number of connections to connect two nodes on average), providing a good estimate of how easily information flows through the network (and therefore of the integration of the network).

#### Global and local efficiency

*Local efficiency* measures the average efficiency of integration within local clusters (e.g. between the neighbours of a given node). *Global efficiency* is the inverse of the characteristic path length and indicates how effectively the information flows across the network.

#### Density

*Density* represents the ratio between the number of actual edges of the network and the number of all possible edges of the network. Before computing density, the network was binarized by removing the weakest 1% of spurious connections, according to the procedure reported in previous studies^42,46^.

Each one of the measures described above (*characteristic path length, global and local efficiency, and density*) were statistically compared between high versus average G*f* groups by using analyses of covariance, where the independent variables where the graph measures, the G*f* group and sex the between-subject factors and the covariates were age and years of education. In this case, we corrected for multiple comparisons by using Bonferroni correction (i.e. dividing the α level of 0.05 by the total number of 24 comparisons (four measures × five frequency band of the functional networks plus one structural connectivity network), resulting in 0.05/24 = 0.002).

### Integration between structural and functional connectivity and G*f*

Finally, we have undertaken an analysis to assess whether we could combine our two modalities (SC and FC) and study such combination in light of the G*f* differences.

First, we computed structural and functional connectivity matrices independently for each participant and frequency band. Then, we computed correlations between the structural connectivity matrix and the functional connectivity ones, independently for each participant and frequency band. Afterwards, we grouped the participants into our two experimental groups (high and average Gf) and tested with ANCOVA (considering age, sex, and education as covariates) whether the two groups differed in terms of similarity between structural and functional connectivity.

## Supplementary Information

Supplementary Information.

## Data Availability

The codes are available at the following link: https://github.com/leonardob92/LBPD-1.0.git, while the multimodal neuroimaging data from the experiment are available upon reasonable request.

## References

[1] Ashton MC, Lee K, Vernon PA, Jang KL (2000). Fluid intelligence, crystallized intelligence, and the openness/intellect factor. J. Res. Pers..

[2] Barbey AK, Koenigs M, Grafman J (2013). Dorsolateral prefrontal contributions to human working memory. Cortex.

[3] Goldstein S, Naglieri JA (2014). Handbook of executive functioning. Handb. Execut. Funct..

[4] Cattell RB (1963). Theory of fluid and crystallized intelligence: A critical experiment. J. Educ. Psychol..

[5] Gray JR, Chabris CF, Braver TS (2003). Neural mechanisms of general fluid intelligence. Nat. Neurosci..

[6] Gardner H, Hatch T (1989). Educational implications of the theory of multiple intelligences. Educ. Res..

[7] Clarke AM, Sternberg RJ (1986). Beyond IQ: A triarchic theory of human intelligence. Br. J. Educ. Stud..

[8] Schneider W, Niklas F, Schmiedeler S (2014). Intellectual development from early childhood to early adulthood: The impact of early IQ differences on stability and change over time. Learn. Individ. Differ..

[9] Santarnecchi E (2017). Network connectivity correlates of variability in fluid intelligence performance. Intelligence.

[10] Criscuolo A, Bonetti L, Särkämö T, Kliuchko M, Brattico E (2019). On the association between musical training, intelligence and executive functions in adulthood. Front. Psychol..

[11] Bonetti L (2018). Auditory sensory memory and working memory skills: Association between frontal MMN and performance scores. Brain Res..

[12] Duncan J, Assem M, Shashidhara S (2020). Integrated intelligence from distributed brain activity. Trends Cogn. Sci..

[13] Bonetti L, Costa M (2016). Intelligence and musical mode preference. Empir. Stud. Arts.

[14] Bonetti L, Costa M (2019). Musical mode and visual-spatial cross-modal associations in infants and adults. Music. Sci..

[15] Bonetti L (2021). Rapid encoding of musical tones discovered in whole-brain connectivity. Neuroimage.

[16] Sternberg RJ (2000). Handbook of Intelligence.

[17] Colom R (2009). Gray matter correlates of fluid, crystallized, and spatial intelligence: Testing the P-FIT model. Intelligence.

[18] Jung RE, Haier RJ (2007). The parieto-frontal integration theory (P-FIT) of intelligence: Converging neuroimaging evidence. Behav. Brain Sci..

[19] Deco G, Tononi G, Boly M, Kringelbach ML (2015). Rethinking segregation and integration: Contributions of whole-brain modelling. Nat. Rev. Neurosci..

[20] Rubinov M, Sporns O (2010). Complex network measures of brain connectivity: Uses and interpretations. Neuroimage.

[21] Basser PJ, Pierpaoli C (2011). Microstructural and physiological features of tissues elucidated by quantitative-diffusion-tensor MRI. J. Magn. Reson..

[22] Góngora D, Vega-Hernández M, Jahanshahi M, Valdés-Sosa PA, Bringas-Vega ML (2020). Crystallized and fluid intelligence are predicted by microstructure of specific white-matter tracts. Hum. Brain Mapp..

[23] Hidese S (2020). Correlation between the wechsler adult intelligence scale-3rd edition metrics and brain structure in healthy individuals: A whole-brain magnetic resonance imaging study. Front. Hum. Neurosci..

[24] Li Y (2009). Brain anatomical network and intelligence. PLoS Comput. Biol..

[25] Van Den Heuvel MP, Stam CJ, Kahn RS, Hulshoff Pol HE (2009). Efficiency of functional brain networks and intellectual performance. J. Neurosci..

[26] Duncan J, Burgess P, Emslie H (1995). Fluid intelligence after frontal lobe lesions. Neuropsychologia.

[27] Roca M (2010). Executive function and fluid intelligence after frontal lobe lesions. Brain.

[28] Woolgar A (2010). Fluid intelligence loss linked to restricted regions of damage within frontal and parietal cortex. Proc. Natl. Acad. Sci. USA..

[29] Wen T, Mitchell DJ, Duncan J (2018). Response of the multiple-demand network during simple stimulus discriminations. Neuroimage.

[30] Assem M, Glasser MF, Van Essen DC, Duncan J (2019). A Domain-general cognitive core defined in multimodally parcellated human cortex. Biorxiv.

[31] Langer N (2012). Functional brain network efficiency predicts intelligence. Hum. Brain Mapp..

[32] Thatcher RW, Palmero-Soler E, North DM, Biver CJ (2016). Intelligence and EEG measures of information flow: Efficiency and homeostatic neuroplasticity. Sci. Rep..

[33] Von Stein A, Sarnthein J (2000). Different frequencies for different scales of cortical integration: From local gamma to long range alpha/theta synchronization. Int. J. Psychophysiol..

[34] Donner TH, Siegel M (2011). A framework for local cortical oscillation patterns. Trends Cogn. Sci..

[35] Mitra A (2016). Human cortical-hippocampal dialogue in wake and slow-wave sleep. Proc. Natl. Acad. Sci. USA.

[36] Groth-Marnat Publisher G, Wiley J (2003). Title: The Handbook of Psychological Assessment.

[37] Lezak MD, Howieson DB, Loring DW, Hannay JH, Fischer JS (2004). Neuropsychological Assessment.

[38] Wechsler D (1997). Wechsler Memory Scale.

[39] Taylor MJ, Heaton RK (2001). Sensitivity and specificity of WAIS-III/WMS-III domographically corrected factor scores in neuropsychological assessment. J. Int. Neuropsychol. Soc..

[40] Hillebrand A, Barnes GR (2005). Beamformer analysis of MEG data. Int. Rev. Neurobiol..

[41] Newman MEJ (2006). Modularity and community structure in networks. Proc. Natl. Acad. Sci. USA..

[42] Fernandes HM (2019). Disrupted brain structural connectivity in pediatric bipolar disorder with psychosis. Sci. Rep..

[43] Cabral J (2014). Exploring mechanisms of spontaneous functional connectivity in MEG: How delayed network interactions lead to structured amplitude envelopes of band-pass filtered oscillations. Neuroimage.

[44] Hindriks R (2015). Role of white-matter pathways in coordinating alpha oscillations in resting visual cortex. Neuroimage.

[45] Brookes MJ (2016). A multi-layer network approach to MEG connectivity analysis. Neuroimage.

[46] Jespersen KV (2020). Reduced structural connectivity in insomnia disorder. J. Sleep Res..

[47] Tzourio-Mazoyer N (2002). Automated anatomical labeling of activations in SPM using a macroscopic anatomical parcellation of the MNI MRI single-subject brain. Neuroimage.

[48] Wechsler D (1997). WAIS-III Administration and Scoring Manual.

[49] Smith SM (2004). Advances in functional and structural MR image analysis and implementation as FSL. Neuroimage.

[50] Woolrich MW (2009). Bayesian analysis of neuroimaging data in FSL. Neuroimage.

[51] Behrens TEJ (2003). Characterization and propagation of uncertainty in diffusion-weighted MR imaging. Magn. Reson. Med..

[52] Jbabdi S, Sotiropoulos SN, Savio AM, Graña M, Behrens TEJ (2012). Model-based analysis of multishell diffusion MR data for tractography: How to get over fitting problems. Magn. Reson. Med..

[53] Taulu S, Simola J (2006). Spatiotemporal signal space separation method for rejecting nearby interference in MEG measurements. Phys. Med. Biol..

[54] Penny W, Friston K, Ashburner J, Kiebel S, Nichols T (2007). Statistical parametric mapping: The analysis of functional brain images. Stat. Paramet. Mapp..

[55] Oostenveld R, Fries P, Maris E, Schoffelen JM (2011). FieldTrip: Open source software for advanced analysis of MEG, EEG, and invasive electrophysiological data. Comput. Intell. Neurosci..

[56] Mantini D (2011). A signal-processing pipeline for magnetoencephalography resting-state networks. Brain Connect..

[57] Brookes MJ (2011). Investigating the electrophysiological basis of resting state networks using magnetoencephalography. Proc. Natl. Acad. Sci. USA.

[58] Power JD, Schlaggar BL, Lessov-Schlaggar CN, Petersen SE (2013). Evidence for hubs in human functional brain networks. Neuron.

