# Supplementary material for: Dissociated brain functional connectivity of fast versus slow frequencies underlying individual differences in fluid intelligence: a DTI and MEG study
Source: Sci Rep. 2022 Mar 18;12:4746. doi: 10.1038/s41598-022-08521-5 (PMC8933399; doi:10.1038/s41598-022-08521-5)
Supplement: Supplementary file 1 — Supplementary Information. [file 41598_2022_8521_MOESM1_ESM.docx]

***Supplementary material for manuscript***

**“Dissociated brain functional connectivity of fast versus slow frequencies underlying individual differences in fluid intelligence: a DTI and MEG study”**

Bruzzone, S.E.P. ^1,5^, Lumaca, M. ^1^, Brattico, E. ^1,4^, Vuust P. ^1^, Kringelbach M.L^1,2,3^, Bonetti L.^2,3,1*^

*^1^Center for Music in the Brain, Department of Clinical Medicine, Aarhus University & The Royal Academy of Music Aarhus/Aalborg, Denmark*

*^2^Centre for Eudaimonia and Human Flourishing, University of Oxford, UK*

*^3^Department of Psychiatry, University of Oxford, Oxford, United Kingdom*

*^4^Department of Education, Psychology, Communication, University of Bari Aldo Moro, Italy*

*^5^Neurobiology Research Unit (NRU), Copenhagen University Hospital Rigshospitalet, Copenhagen, Denmark*

**Corresponding author*

***Supplementary Tables***

The supplementary tables can be found at the following link: <https://drive.google.com/drive/folders/1OSdaAbNoCO5zpQJrCYRKiHWoYgiEI882?usp=sharing>

***Table ST1. Degree***

*Brain areas (ROIs) one standard deviation above (or below, as depicted by dash line in* ***Figure 3****) the mean degree. The ROIs with the strongest values are the ones that had the highest difference in terms of degree when comparing High versus Average Gf groups (i.e. highest values correspond to ROIs that had a stronger degree for High versus Average Gfs). These areas are depicted in* ***Figure 3*** *in the brain templates.* ***Table ST1*** *reports ROIs independently for DTI and the five frequency bands from MEG.*

***Table ST2. Community structure***

*Brain areas (ROIs) reported in the different communities (modules) outputted by the modularity algorithm that we used in the study (Newman, 2006). The community structures are reported independently for DTI and the five frequency bands from MEG. These community structures are depicted in brain templates in* ***Figure 5****.*

***Table ST3. Reversed segregation coefficient***

*Brain areas (ROIs) one standard deviation above (or below, as depicted by dash line in* ***Figure 4****) the mean segregation coefficient. In this case, the ROIs with the smallest values are the ones that had the highest difference in terms of segregation coefficient when comparing High versus Average Gf groups (i.e. smallest values correspond to ROIs that had more inter- than intra-community connections for High versus Average Gfs). These areas are depicted in* ***Figure 4*** *in the brain templates.* ***Table ST3*** *reports ROIs independently for DTI and the five frequency bands from MEG.*
